# Increased of serum high-mobility group box chromosomal protein 1 correlated with intestinal mucosal barrier injury in patients with severe acute pancreatitis

**DOI:** 10.1186/1749-7922-9-61

**Published:** 2014-12-17

**Authors:** Gui-fang Xu, Ming Guo, Zhi-qiang Tian, Guo-zhong Wu, Xiao-ping Zou, Wei-jie Zhang

**Affiliations:** Department of Gastroenterology, Nanjing Drum tower Hospital, The Affiliated Hospital of Nanjing University Medical School, Nanjing, China; Department of Gastroenterology, Drum Tower Clinical College of Nanjing Traditional Chinese Medical University, Nanjing, China; Department of General Surgery, 101st Hospital of People’s Liberation Army, Wuxi, China; Department of Emergency, Nanjing Drum tower Hospital, The Affiliated Hospital of Nanjing University Medical School, Nanjing, China

**Keywords:** Severe acute pancreatitis, Intestinal mucosal barrier, Injury, High-mobility group box chromosomal protein 1 (HMGB1), Inflammation, Infection

## Abstract

**Background:**

Secondary infections are the leading cause of death in patients with severe acute pancreatitis (SAP). The gut represents the main source of pancreatic contamination and related septic complications. High-mobility group box chromosomal protein 1 (HMGB1) was recently identified to play an important role in the SAP intestinal mucosal barrier dysfunction.

**Objective:**

To investigate the correlation of high-mobility group box 1 (HMGB1) with intestinal barrier injury and infections in patients with severe acute pancreatitis (SAP).

**Methods:**

The serum levels of HMGB1, amylase, lipase, and biochemical indicators were measured in 80 patients with SAP at the time of admission. Furthermore, relationship between their serum HMGB1 levels and intestinal barrier injury, infection and other clinical factors were analyzed.

**Results:**

The mean value of serum HMGB1 levels was significantly higher in patients with SAP (6.02 ± 2.42 ng/mL) than that in healthy volunteers (1.87 ± 0.63 ng/mL). Serum HMGB1 levels were significantly positively correlated with the Ranson score. The HMGB1 levels were higher in patients with infection during the clinical course, the HMGB1 levels in non-survivors were higher than those in survivors, and positively correlated with DAO activity, L/M ratio, the concentration of endotoxin (R = 0.484, *P* <0.01).

**Conclusions:**

HMGBl level of patients with severe acute pancreatitis was significantly increased, and can be used as an important indicator to determine the intestinal barrier dysfunction and infection.

Severe acute pancreatitis (SAP) has high mortality, but multiple and timely interventions can improve survival [1]. In patients with SAP, SIRS-related gut hypoperfusion and mucosal damage may occur early, and may contribute to clinical worsening. It has been showed that in SAP early gut mucosal damage severity correlates with clinical severity, as well as with the development of secondary infections [2].

High-mobility group box chromosomal protein 1 (HMGB1), originally discovered as a nuclear DNA-binding protein, was recently identified as a potent proinflammatory and late-acting mediator of endotoxin lethality [3]. Experimental models of SAP identified HMGB1 as a key contributor to gut barrier dysfunction, and found a significant correlation between HMGB1 serum levels and the severity of intestinal mucosal injury [4].

In clinical studies of SAP, HMGB1 acts as an early marker of disease worsening, by reflecting pancreatic tissue injury and inflammation. Serum levels of HMGB1 of SAP patients on admission are significantly higher than in healthy controls, and significantly correlate with the severity of the disease [5]. However, the relationship between HMGB1 and gut mucosal injury has not been investigated in a clinical setting.

In this study, we aim to clarify whether HMGB1 is correlated with intestinal mucosal barrier injury and infection of SAP; and the relationships between their serum HMGB1 levels and various clinical factors for SAP were also analyzed.

## Materials and methods

### Patients and samples

Serum samples were obtained from 10 healthy volunteers and patients with SAP in our department between October 2003 and April 2007. Only patients at the time of admission within the initial 72 hours was admitted. The samples were stored at -80°C until they were assayed. The diagnosis of acute pancreatitis requires two of the following three features: (1) abdominal pain consistent with acute pancreatitis (acute onset of a persistent, severe, epigastric pain often radiating to the back); (2) serum lipase activity (or amylase activity) at least three times greater than the upper limit of normal; and (3) characteristic findings of acute pancreatitis on contrast-enhanced computed tomography (CECT) and less commonly magnetic resonance imaging (MRI) or transabdominal ultrasonography [6].

The classification defines three degrees of severity: mild acute pancreatitis, moderately severe acute pancreatitis, and severe acute pancreatitis [6, 7]. Terminology that is important in this classification includes transient organ failure, persistent organ failure, and local or systemic complications (Table 1) [6]. Transient organ failure is organ failure that is present for <48 h. Persistent organ failure is defined as organ failure that persists for >48 h.

**Table 1:** **Grades of severity of acute pancreatitis**

**Mild acute pancreatitis**	
▸ No organ failure	
▸ No local or systemic complications	
**Moderately severe acute pancreatitis**	
▸ Organ failure that resolves within 48 h (transient organ failure) and/or	
▸ Local or systemic complications without persistent organ failure	
**Severe acute pancreatitis**	
▸ Persistent organ failure (>48 h)	
– Single organ failure	
– Multiple organ failure	

Ref [6] Banks PA, et al. Gut. 2013; 62(1):102-111.

The presence of infection can be presumed when there is extraluminal gas in the pancreatic and/or peripancreatic tissues on CECT or when percutaneous, image-guided, fine-needle aspiration (FNA) is positive for bacteria and/or fungi on Gram stain and culture [8].

The following baseline and outcome variables were recorded for each patient on admission: age, gender, etiology, the coexisting condition, acute physiology and chronic health evaluation II (APACHE II) score, Ranson score (>3 as high and ≤ 3 as low Ranson group), CT severity index (CTSI)-which is a 10-point scoring system derived by assessing the degree of pancreatic and peripancreatic inflammation (0 to 2 points), the presence and number of peripancreatic fluid collections (0 to 2 points), and the presence and degree of pancreatic parenchymal non-enhancement or necrosis (0 to 6 points) [9], the extent of pancreatic necrosis, the multiple organ dysfunction score (MODS), the mortality, and the length of hospital stay. The following items were also recorded at enrollment: the CECT before study entry; leukocyte count and neutrophil percentage; blood biochemical parameters, such as: the serum levels of C-reactive protein (CRP), amylase, procalcitonin (PCT), and HMGB1 were all evaluated. The serum HMGB1 levels were determined, and the relationships with sex, etiology, pancreatic necrosis, severity, blood biochemical parameters on admission, organ dysfunction and infection during the clinical course, and prognosis were also analyzed.

The study was approved by the ethics committees of the Nanjing Drum Tower Hospital and informed consent was obtained from each patient.

### Serum concentrations of high-mobility group box chromosomal protein 1

HMGB1 was measured by the commercially available HMGB1 ELISA Kit II (SHINO-TEST Corporations, Kanagawa, Japan) [10]. Briefly, in the wells coated with anticalf HMGB1 monoclonal antibody, samples to be measured or standards are incubated for 24 hours at 37°C. After washing the well for 5 times, a peroxidase-conjugated anti-HMGB1 monoclonal antibody was added into the microwell and incubated for 120 minutes at room temperature. After washing the well for 5 times, 100 KL of luminogen was added into the microwell and incubated for 30 minutes at room temperature. A stop solution was added to each well to terminate the enzyme reaction and to stabilize the developed color. The optical density of each well was measured at 450 nm using a microplate reader. The results were calculated using a calibration curve prepared from standards.

### The plasma diamine oxidase (DAO) activity

DAO activity was assayed according to the modified method of Nobumichi et al. [11]. In the final volume of 3.8 ml, the assay mixture contained 3ml of phosphate buffer (0.2 M, pH 7.2), 0.1 ml (4 μg) of horseradish peroxidase solution (Sigma), 0.1 ml of *o*-dianisidine methanol solution (500μg of *o*-dianisidine purchased), 0.5 ml plasma; 0.1 ml of substrate solution (175 μg of cadaverine dihydrochloride from Sigma). This sample was incubated for 30 min at 37°C, and DAO activity was measured as the absorbance at 436 nm.

### Intestine mucosal permeability

This was assessed by the lactulose–mannitol (L/M) ratio in urine. Patients were administered orally 60 ml solution (consisting of 10 g lactulose and 5 g mannitol) after evacuating the urinary bladder in the morning, and their urine was collected for the subsequent 6 h. During this period the patients were not permitted any oral intake of food or water. The urine volume was measured and the concentration of lactulose and mannitol were determined according to the method of Sörensen and Proud [12].

### The plasma endotoxin level

This was detected by the end-point chromogenic method based on the activation of a limulus amoebocyte lysate. Heparin sodium (20U) was added to each blood sample (3ml), and centrifuged at 300 rpm/min for 10 min to obtain plasma. Each sample was diluted at 1:4 with pyrogen-free water and heated at 100°C for 8 min to inhibit the interfere factors with limulus assay. The manipulation was done according to the instructions in the kits provided by Sigma.

### Statistical analysis

Statistical analysis was performed using SPSS version 16.0 software (SPSS, Inc., Chicago, IL, U.S.A.). Unless test for normality of data distribution has been made, the results are given as medians with ranges and interquartile ranges (IQR). For comparison of the two groups, *χ*^2^-analysis or Fisher’s exact test were used when appropriate for qualitative data, and Student’s *t*-test for quantitative data. Pearson correlation analysis was performed to assess the correlations between HMGB1 and the continuous variables, and Spearman correlation was performed to assess the correlations between HMGB1 and the non-continuous variables. *P* value less than 0.05 was considered statistically significant.

## Results

### Patient’s characteristics

The study group consisted of 80 patients with (47 men and 33 women). A total of 80 SAP patients were included in this study. Forty-eight patients (58.8%) were male, and the median age of the patients was 43 years (19–89 years). The median time interval between onset and admission was 32 hours (2–71 hours). The level of serum amylase was 1900 U/L (430-3100 U/L). The CRP and PCT level was 11.73 mg/dL (0.79-44.08 mg/dL) and 4.28 ng/mL (0.01-166.69 ng/mL), respectively. The median length of hospital stay was 20 days (13-64 days), and the mortality rate was 15 out of 80 patients (18.8%). The clinical characteristics, etiology, severity and clinical outcomes of patients were in Table 2.

**Table 2:** **The clinical features, etiology, severity and clinical outcomes of the patients with SAP**

Patients characteristics	No. of cases	Percentage
**Sex**		
Male	47	58.8
Female	33	41.2
**Comorbidities**		
Diabetes mellitus	7	8.8
Hypertension	9	11.3
Chronic kidney disease	5	6.3
Other	9	11.3
No comorbid condition	50	62.5
**Etiology**		
Biliary	47	58.8
Alcoholic	19	23.8
Idiopathic	9	11.3
Hypertriglyceridemia	3	3.8
Post-ERCP	2	2.5
**Severity**		
APACHE II		
≥8	60	75
≤7	20	25
CT severity index		
≥4	70	87.5
≤3	10	12.5
Ranson score		
>3	64	80
≤3	16	20
**Complications**		
Secondary infections	23	28.8
MODS	60	75
**Mortality**	15	18.8

**Table 3:** **The Intestine mucosal barrier function between healthy volunteers and patients with SAP**

Parameter	Healthy volunteer	Severe acute pancreatitis	***P*** value
	(n = 10)	(n = 80)	
**DAO (IU/ml)**	0.370 ± 0.16	3.45 ± 1.67	<0.001
**L/M**	0.03 ± 0.01	0.26 ± 0.09	<0.001
**Endotoxin (EU/ml)**	0.0870 ± 0.019	0.3613 ± 0.16	0.01

### Serum high-mobility group box chromosomal protein 1 levels in patients with severe acute pancreatitis

The mean value of serum HMGB1 levels in patients with SAP at the time of admission (within 72 hours after the onset) was 6.02 ± 2.42 ng/mL and was significantly higher than that in healthy subjects (1.87 ± 0.63 ng/mL).

### Relationship with blood biochemical parameters on admission

**Figure 1:**
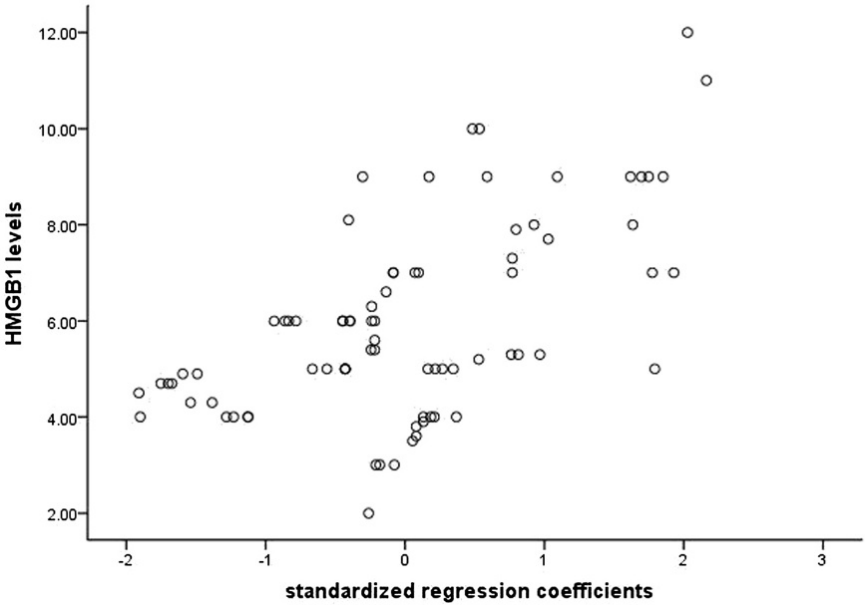
**Correlation of serum high-mobility group box chromosomal protein 1 levels with blood biochemical parameters on admission in patients with severe acute pancreatitis.**

Correlation efficient of serum HMGB1 levels with various blood biochemical parameters on admission was investigated. Procalcitonin, total bilirubin and C-reactive protein were significantly positively correlated with serum HMGB1 levels (R = 0.393, *P* < 0.05, Figure 1). We could not find the significant correlation between other parameters (amylase, etc.) and serum HMGB1 levels. The value of HMGB1 versus amylase, lipase, ALT, and AST was R = 0.118, *P* = 0.297; R = 0.175, *P* = 0.120; R = 0.122 p = 0.286, and R = 0.156 p = 0.174, respectively.

### Relationship with severity score on admission

**Figure 2:**
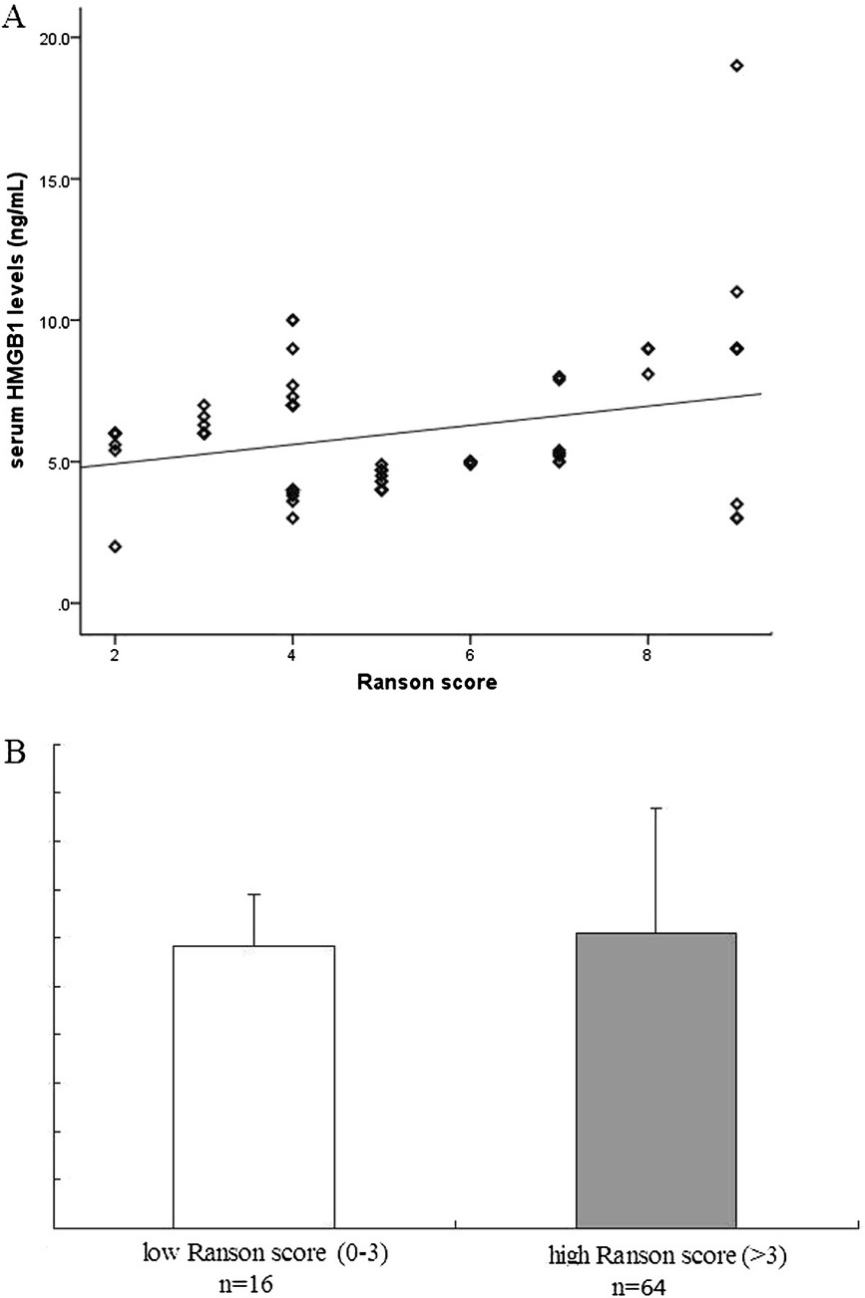
**A, Correlation of serum HMGB1 levels with Ranson scores on admission. B, A comparison between serum levels of HMGB1 in high (>3) and low (<3) Ranson score groups.**

Serum HMGB1 levels were positively correlated with Ranson score (R = 0.297; Figure 2A) and were significantly higher in the high Ranson score group (6.1 ± 2.6 ng/mL) than those in the low Ranson score group (5.8 ± 1.1 ng/mL), (*p* = 0.032 Figure 2B), whereas serum HMGB1 levels were not correlated with CT severity index (R = 0.017) or APACHE II score (R = 0.058).

### Relationship with intestine mucosal barrier function

The plasma DAO activity and serum endotoxin levels were significantly higher were significantly higher in patients with SAP than that of healthy control (*P <* 0*.*01). The urinary lactulose concentrations were significantly higher in SAP patients than in healthy controls (*P* < 0.01). There is no significant difference in mannitol excretion between two groups. The lactulose to mannitol ratio in the SAP group is significantly higher than that of healthy groups (*P* < 0.01) (Table 3).

**Figure 3:**
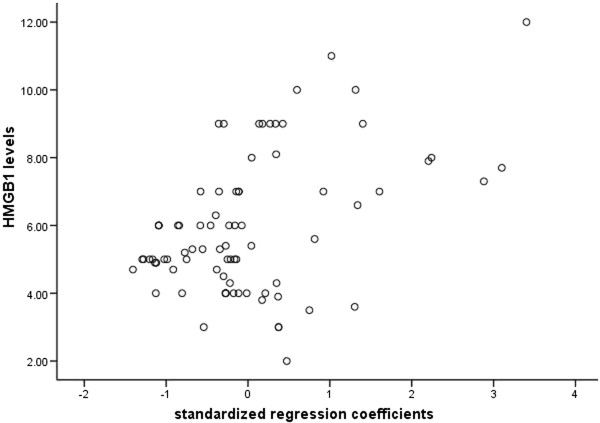
**Correlation efficient of serum HMGB1 levels with intestinal mucosal barrier parameters (plasma DAO activity, serum endotoxin, urinary L/M ratio).**

Correlation efficient of serum HMGB1 levels with intestinal mucosal barrier parameters on admission was investigated, and found that plasma DAO activity, serum endotoxin, urinary lactulose and mannitol ratio (L/M) were significantly positively correlated with serum HMGB1 levels (R = 0.484, *P* < 0.01, Figure 3).

### Relationship between infection during hospitalization with the intestine mucosal barrier

The plasma DAO activity, serum endotoxin and lactulose to mannitol ratio levels were higher in patients with infection (2.90 ± 1.37 and) than those without infection, but only serum endotoxin (0.33 ± 0.13 vs 0.44 ± 0.21, *p* = 0.001) was significant higher, the plasma DAO activity (2.90 ± 1.37 vs 4.82 ± 1.57, *p* = 0.234) and L/M (0.23 ± 0.07 vs 0.34 ± 0.08, *p* = 0.275) were not significant.

### Relationship with infection during hospitalization and prognosis

**Figure 4:**
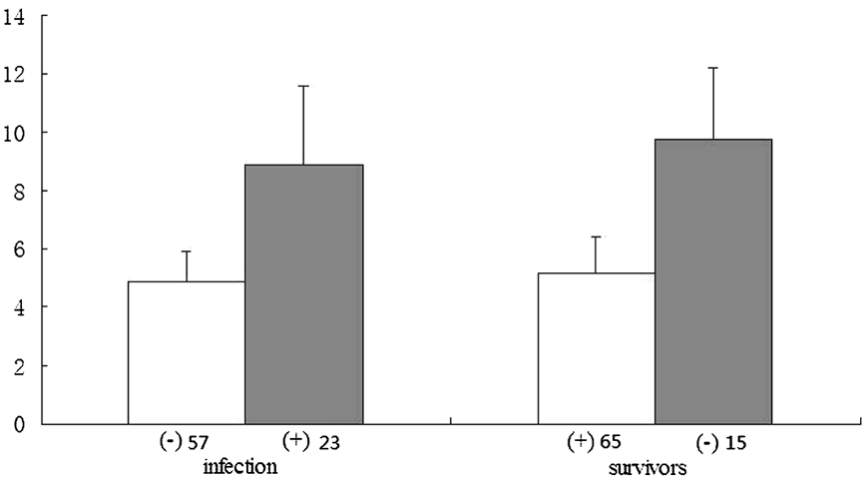
**Relationship of serum HMGB1 levels with infection and prognosis. Infection was defined as described under Materials and Methods.**

Serum HMGB1 levels in patients with infection (8.9 ± 2.47 ng/mL) were significantly higher than those in patients without infection (4.9 ± 1.04 ng/mL). Serum HMGB1 levels in non-survivors (9.7 ± 2.68 ng/mL) were higher than those in survivors (5.2 ± 1.3 ng/mL), but there was no significant difference (Figure 4).

## Discussion

In this study, we evaluated serum HMGB1 concentrations in 80 patients with SAP and compared them to the concentrations in healthy control subjects. Furthermore, correlation between serum HMGB1 levels and blood biochemical parameters, intestinal mucosa barrier, and infections in patients with SAP were also analyzed.

We identified that the concentration of serum HMGB1 was higher in patients with SAP than in normal healthy subjects, and it can be used as an important indicator to determine the intestinal barrier dysfunction and infection in clinical setting.

Blood biochemical parameters, such as C-reactive protein (CRP) and procalcitonin (PCT) have been considered to evaluate the evolution of infections and sepsis in critically ill patients. However, both proteins are also induced during non-infectious causes of systemic inflammation and in patients with organ dysfunction [13]. Total bilirubin combined with serum glutamate oxaloacetic transaminase (AST) or glutamate pyruvic transaminase (ALT) are predicators of liver dysfunction, especially in biliary acute pancreatitis. In the results obtained, we have demonstrated that serum HMGB1 levels were significantly positively correlated with PCT, CRP and total bilirubin on admission. These results suggest that HMGB1 may be closely related to the aggravation of SAP in the early phase and that increase of serum HMGB1 may reflect tissue injury and inflammation.

DAO is found almost exclusively in the small intestine, its activity is closely correlated with villi height, nucleic acid, and protein synthesis of intestinal mucosal cells. The intestinal tissue and serum levels of DAO have been used as an indicator of the integrity and functional mass of the intestinal mucosa, and also lactulose/mannitol seems to be an inexpensive and quite reliable method for interpreting ntestinal permeability. In this study, we found the activity of plasma DAO; the levels of plasma endotoxin and urinary L/M ratio were all elevated markedly, and this phenomenon was found in patients with infection, but only serum endotoxin was significant higher than those without infection.

HMGB1 act as a late-acting mediator of development of ileal mucosal hyperpermeability and increased bacterial translocation, promotes alterations in gut barrier function by increasing the permeability in enterocytic monolayers and increasing bacterial translocation to lymph nodes [14]. It has been suggested that stabilization of intestinal integrity reduced pancreatic infections in experimental and clinical pancreatitis, breakdown of the gut mucosal barrier resulted in the passage of luminal bacteria and endotoxin into the systemic circulation [2, 4, 15]. Therefore, improvement of increased gut permeability is a key step for control of the infectious complications. Rats with SAP showed an increased intestinal permeability when compared with the controls, and the subsequent endotoxin translocation was also confirmed [16]. Consistent with our observations, we observed HMGB1 levels were higher in patients with secondary infection and non-survivors during the clinical course. We have also obtained that the HMGB1 levels were higher in patients with intestinal mucosal barrier injury for the first time. The curculated HMGB1 concentrations were remarkably increased in patients with SAP and correlated with the severity of intestinal barrier dysfunction. Results obtained here with these observations raise the possibility that blockade of HMGB1 in the early phase may be useful as a new therapeutic option against the inflammation and gut mucosal injury in SAP [17].

In this study, we have shown that serum HMGB1 levels were significantly increased in patients with SAP, and for the first time in clinical setting, we found circulating HMGB1 were correlated with the severity of intestinal barrier dysfunction. Such results give direct evidence HMGB1 might be the key factor causing morphologic destruction of small intestinal mucosal barrier. These results suggest that HMGB1 may play a pivotal role in the pathogenesis of SAP and that HMGB1 may act as a promoter and early marker of gut mucosal injury.
